# Compressed Sensing‐Accelerated Free‐Breathing Liver MRI at 7 T

**DOI:** 10.1002/nbm.70047

**Published:** 2025-04-28

**Authors:** Mitra Tavakkoli, Bobby A. Runderkamp, Matthijs H. S. de Buck, Gustav J. Strijkers, Michael D. Noseworthy, Aart J. Nederveen, Matthan W. A. Caan, Wietske van der Zwaag

**Affiliations:** ^1^ Department of Electrical and Computer Engineering McMaster University Hamilton Ontario Canada; ^2^ Imaging Research Centre St. Joseph's Healthcare Hamilton Hamilton Ontario Canada; ^3^ Department of Radiology and Nuclear Medicine, Amsterdam University Medical Center University of Amsterdam Amsterdam the Netherlands; ^4^ Spinoza Centre for Neuroimaging Royal Netherlands Academy for Arts and Sciences (KNAW) Amsterdam the Netherlands; ^5^ Computational and Cognitive Neuroscience and Neuroimaging Netherlands Institute for Neuroscience, KNAW Amsterdam the Netherlands; ^6^ Department of Biomedical Engineering and Physics, Amsterdam University Medical Center University of Amsterdam, Amsterdam Cardiovascular Sciences Amsterdam the Netherlands; ^7^ Department of Medical Imaging McMaster University Hamilton Ontario Canada; ^8^ School of Biomedical Engineering McMaster University Hamilton Ontario Canada

**Keywords:** 7 T MRI, abdomen, compressed sensing, free‐breathing, high‐resolution, liver

## Abstract

Ultra‐high field MRI facilitates imaging at high spatial resolutions, which may become important for detailed anatomical and pathological assessment of the human liver. Therefore, we aimed to advance structural liver imaging at 7 T by implementing a high‐resolution, phase‐shimmed, free‐breathing liver scan. Six healthy participants underwent liver MRI scans at 7 T, utilizing an eight‐channel parallel transmission system for phase shimming. B_0_ mapping and Fourier phase‐encoded dual refocusing echo acquisition mode (PE‐DREAM) multichannel B_1_^+^ mapping were performed during breath‐holds at expiration. Prospectively undersampled golden‐angle pseudo‐spiral k‐space data were acquired under free breathing, enabling retrospective respiratory binning using self‐gating. Post‐binning, the simultaneous autocalibrating and k‐space estimation (SAKE) algorithm was employed for interpolation of a center of k‐space area, prior to estimation of receive coil sensitivity maps. Image reconstruction was performed on expiration‐phase data using compressed sensing, optimizing image quality by evaluating various regularization factors and numbers of respiratory bins. Finally, N4BiasFieldCorrection was applied to the resulting images. Expiration‐phase image reconstruction using four bins and regularization factor values of 10^−2.5^ (1.50 mm) and 10^−2.33^ (1.35 mm) were found to optimize the tradeoff between sharpness, SNR, and artifacts. The optimized protocol facilitated clear visualization of the liver, blood vessels, and surrounding structures at isotropic resolutions of 1.50 and 1.35 mm in 3.5 min, without B_1_^+^ inhomogeneity effects in the shimmed liver region. A comparison between low‐resolution fully sampled free‐breathing (3.5 min) and breath‐hold (19 s) acquisitions demonstrated comparable sharpness and SNR. To compare the 7 T data with 3 T MRI, 3 T scans were performed for two participants. 3 T reconstructions were done similarly to 7 T, excluding N4BiasFieldCorrection. Scan‐specific regularization optimization was performed for fair comparison. Compared to 3 T, 7 T showed superior vascular contrast with inflow effects not observed at 3 T. Fold‐over artifacts were present in 3 T scans but were minor at 7 T. 3 T and 7 T provided comparable results, with a much higher RF channel count at 3 T. In conclusion, high‐resolution expiration‐phase liver imaging at 7 T with homogeneous signal can be successfully achieved using a phase‐shimmed, free‐breathing protocol with a golden‐angle pseudo‐spiral sampling pattern technique and respiratory self‐gating. This approach allows detailed anatomical depiction without the limitations of breath‐holding, representing a significant advancement in ultra‐high field abdominal MRI.

## Graphical abstract

In this study, compressed‐sensing acceleration, respiratory binning and eight‐channel phase shimming were combined to enable free‐breathing liver MRI at 7 Tesla. Three‐dimensional gradient‐echo liver images with 1.35 mm isotropic resolution could be acquired in 3.5 min without B_1_^+^ inhomogeneity effects, allowing detailed anatomical depiction without the limitations of breath holding.

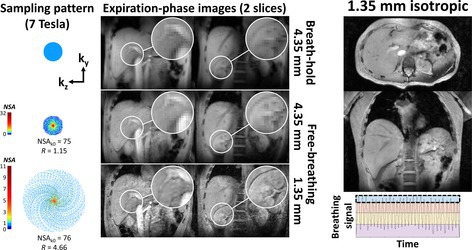

**Abbreviations**
AP: anterior–posterior
BH: breath‐hold
CS: compressed sensing
FB: free‐breathing
FH: foot‐head
GRE: gradient‐recalled echo
IVC: inferior vena cava
MLV: maximum local variation
MTF: modulation transfer function
NSA: number of signal averages
PE‐DREAM: phase‐encoded dual refocusing echo acquisition mode
PICS: parallel imaging and compressed sensing
PROUD: prospective undersampling in multiple dimensions
PSF: point spread function
pTx: parallel transmit
RL: right–left
ROI: region of interest
RSG: respiratory self‐gating
SAKE: simultaneous autocalibrating and k‐space estimation
TIAMO: time interleaved acquisition of modes
UHF: ultrahigh field
VOI: volume of interest

## 1 Introduction

Ultrahigh field (UHF; B_0_ ≥ 7 T) MRI offers significantly increased SNR compared to lower‐field MRI systems [1]. This higher SNR can be traded for higher spatial resolution, as has been demonstrated for numerous applications in the brain [2]. Likewise, 7 T MRI has the potential to improve SNR, image sharpness, and tissue contrast in abdominal imaging compared to 3 T or 1.5 T [3, 4].

With a large FOV for the abdomen and high‐resolution imaging, the matrix size to be filled increases dramatically and, consequently, abdominal scan durations quickly exceed the practical limits of breath‐holding. Patients, in particular, may find breath‐holding more challenging to perform, potentially jeopardizing image quality in a clinical target group. Respiratory binning can be used to obtain images uncorrupted by motion simply by selecting the timepoints during which data are to be combined for reconstruction [5]. In respiratory self‐gating [6], respiratory motion information is extracted directly from the acquired k‐space data, requiring no extra navigator acquisitions or physiological monitoring equipment, which further adds to patient comfort and compliance. Similar approaches have been adopted in cardiac MRI, where the additional dimension of cardiac motion has to be contended with [7]. To cope with the unpredictable nature of the resulting acquisition pattern and to achieve high‐resolution images in clinically acceptable scan times, self‐gating should be combined with robust sampling techniques such as golden‐angle radial or spiral k‐space undersampling patterns, as in XD‐GRASP [8]. Compressed sensing (CS) reconstructions are highly suited for reconstructing the resulting randomly undersampled data [9]. Free‐breathing (FB), radially sampled, CS reconstructed anatomical data have previously been acquired to, e.g., study volumetric fat/water fractions [10].

However, the increased B_1_^+^ inhomogeneities associated with 7 T become particularly troublesome when imaging large FOVs, such as the human abdomen [3, 11, 12]. In small FOVs like the brain and extremities, acceptable image quality can be achieved even with relatively standard quadrature coil designs [13]. The signal voids associated with B_1_^+^ inhomogeneities in the abdomen, however, are prohibitive without appropriate acquisition strategies. Recent advancements have demonstrated that parallel transmission (pTx), using eight or more individually driven transmit coil elements, can substantially improve image homogeneity in the abdomen at UHF [14]. Using one or multiple [15] phase‐shimmed acquisitions (static pTx) or more advanced excitation k‐space [16] trajectories (dynamic pTx), improved image homogeneity has been achieved in both the heart [17, 18] and liver [19, 20] at 7 T and above [21]. Moreover, for successful application of pTx, availability of accurate multichannel B_1_^+^ maps is required. Recently, Runderkamp et al. [20] showed that good image homogeneity can be achieved in 3D breath‐hold whole‐liver gradient‐echo (GRE) acquisitions at 7 T using a phase shim calculated based on Fourier phase‐encoded dual refocusing echo acquisition mode (PE‐DREAM) [22] multichannel B_1_^+^ maps.

In this work, parts of which have previously been presented at the annual meeting of the ISMRM [23], we develop a tailored scanning and reconstruction protocol for high‐resolution expiration‐phase structural liver imaging at 7 T with high signal homogeneity throughout the organ. To this end, we implemented a phase‐shimmed, FB liver scan with a golden‐angle pseudo‐spiral CS‐accelerated acquisition that allows retrospective respiratory binning.

## 2 Methods

### 2.1 MRI Acquisition

For this study, six healthy participants (two female; 25–43 years, BMI range 20.7–23.6 kg/m^2^, body‐circumference range 74–97 cm) were scanned after providing written informed consent. The body circumference was measured by drawing a contour around the abdomen in an axial slice positioned at the largest axial section of the liver. All experiments in this study adhered to the guidelines of the local Medical Ethical Committee and were performed in accordance with the declaration of Helsinki. MRI measurements were performed using a 7 T MRI scanner (Philips Healthcare, Best, The Netherlands) equipped with a whole‐body gradient coil (40 mT/m maximum gradient strength, 200 T/m/s slew rate) and eight 2‐kW peak RF power amplifiers. The eight‐channel pTx system was used in conjunction with a 30‐cm long, eight‐channel transmit‐receive dipole antenna body array [24].

After abdominal localizer scans, second‐order B_0_ shimming was performed based on a dual‐echo 3D‐GRE acquisition. Next, Fourier PE‐DREAM [22] B_1_^+^ mapping was performed using 13 phase‐encoding steps acquired in separate breath‐holds at expiration [20]. Other scan settings for B_0_ and B_1_^+^ mapping were chosen as described by Runderkamp et al. [20]. Based on those B_1_^+^ maps, the eight‐channel phase offsets, defined by channel‐specific complex weights $a_{c}$ with equal amplitude ($\left|a_{c}\right|$ = 1), were optimized by minimizing the cost function $\mathrm{SD}\left(\left|\sum_{c=1}^{8}S_{c}a_{c}\right|\right)/\text{mean}{\left(\left|\sum_{c=1}^{8}S_{c}a_{c}\right|\right)}^{2}$ [20]. Here, $S_{c}$ is the B_1_^+^ or “transmit sensitivity” map of transmit channel *c*, expressed as a percentage of the nominal flip angle of the STEAM preparation pulses [25]. B_0_‐ and phase‐shim values were calculated for the same volume of interest (VOI) containing the entire liver, which was drawn manually on a slice‐by‐slice basis using the magnitude image of the first echo of the B_0_ map acquisition. MRCodeTool version 1.5.15 (Tesla DC, Zaltbommel, The Netherlands) was used to draw the VOI and calculate the B_0_‐ and phase‐shim values. Moreover, manual RF power calibration was performed for each participant to acquire a mean flip angle over the participant‐specific VOI close to the Ernst angle of the liver at 7 T, cos^−1^(exp(−4.6/1362)) ≈ 4.71°, assuming TR = 4.6 ms and liver T_1_ = 1362 ms [26].

**FIGURE 1:**
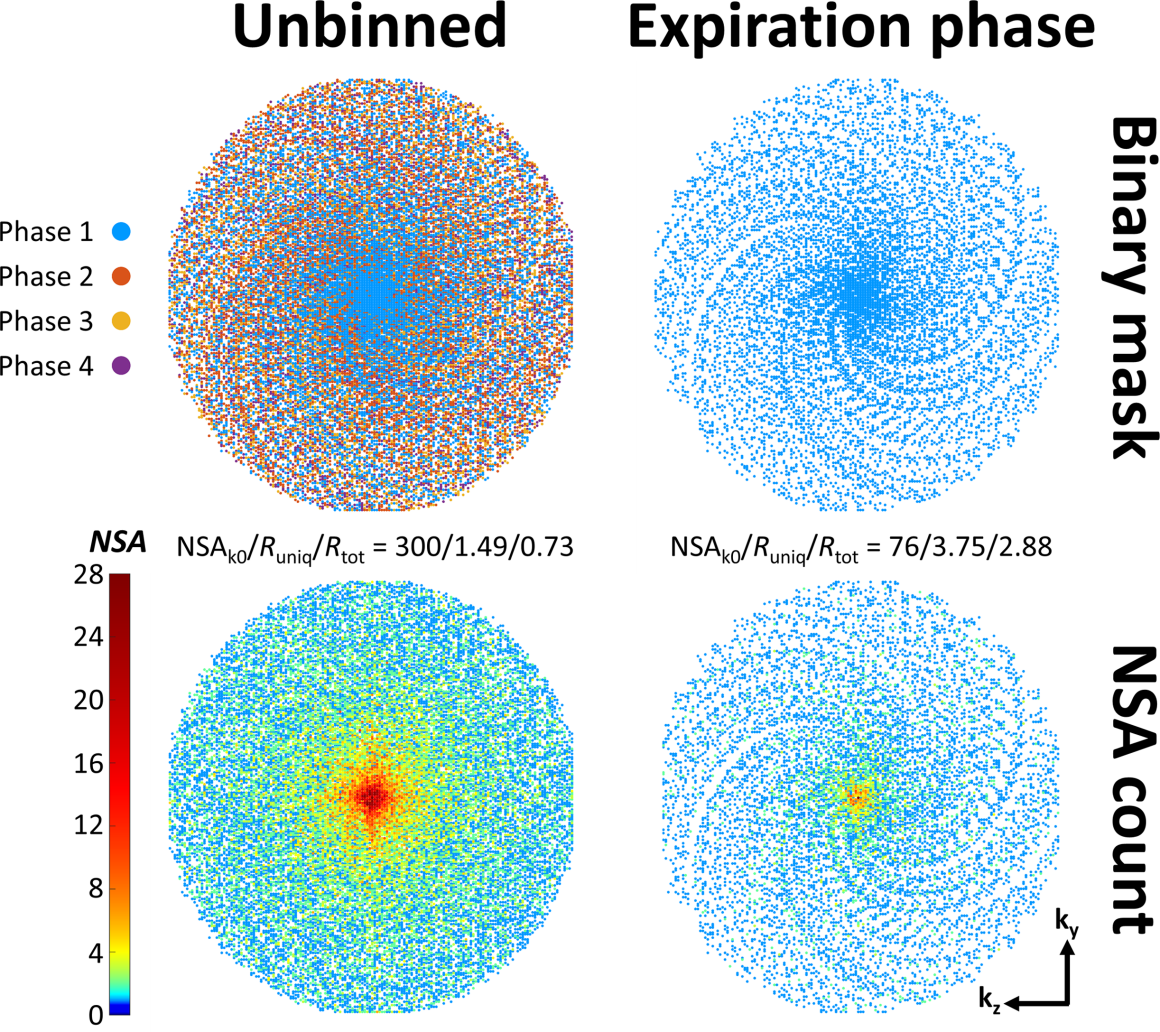
Pseudo‐spiral sampling patterns of the unbinned (left) and expiration‐phase (right) k‐space. Each point represents a fully sampled k_x_ readout line. The binary mask is shown in the top row, while the bottom row illustrates the resulting variable‐density averaging pattern. In this example, four respiratory phases were used for binning of a 1.50 mm scan. NSA_k0_: number of signal averages of the k‐space center.

T_1_‐weighted RF‐spoiled 3D‐GRE scans were subsequently acquired using these B_0_‐ and phase‐shim values. The preceding B_0_ and phase shimming using expiration‐phase breath‐holds ensures that data for the targeted expiration‐phase reconstruction are acquired under stable shimming conditions. The FB GRE acquisitions in this study were prospectively undersampled using the PROspective Undersampling in multiple Dimensions (PROUD) software patch (https://mriresearch.amsterdam/software/). A tiny golden‐angle [27] pseudo‐spiral sampling pattern was used with spiral arms of 25 points each (Figure 1). To allow for respiratory binning, the k‐space center was sampled every 150 lines, i.e., 690 ms, to adequately sample the respiratory cycles. Readouts were used along the foot–head (FH) direction to obtain profiles that crossed the liver dome, capturing respiratory motion. After binning, data from the expiration‐phase bin were used for image reconstruction (see subsection Image Reconstruction). This led to pseudo‐random (under)sampling of k‐space with a decreasing number of signal averages (NSA) from the center to the periphery of k‐space (Figure 1, bottom row). The undersampling factor can be defined as R_uniq_ = (N_ky_ × N_kz_ × π/4)/N_uniq_, where N_ky_ and N_kz_ are matrix sizes along k_y_ and k_z_, the factor π/4 corrects for the elliptical shutter that was assumed to be present in the fully sampled case, and N_uniq_ is the total number of uniquely sampled k_y_‐k_z_ locations, i.e., disregarding multiple averages of the same k_y_‐k_z_ location. Moreover, the acceleration factor can be defined as R_tot_ = (N_ky_ × N_kz_ × π/4)/N_tot_, where N_tot_ is the total number of sampled k‐lines, including any averages of the same k_y_‐k_z_ location. Figure 1 illustrates the effect of binning and continuous pseudo‐spiral sampling on the k‐space pattern, for the example of an acquisition with spatial resolution of 1.50 × 1.50 × 1.60 mm^3^ reconstructed into four bins.

For Participant 1, an experiment was conducted to show the feasibility of changing a breath‐hold (BH) acquisition to a FB acquisition, and the obtainable increase in resolution as a result. First, a fully sampled breath‐hold scan (expiration) was performed with spatial resolution (FH × RL × AP) of 4.35 × 4.35 × 4.35 mm^3^ (“BH 4.35 mm”), FOV = 300 × 388 × 265 mm^3^, matrix size = 69 × 89 × 61, phase‐encoding directions right–left (RL) and anterior–posterior (AP), coronal slice orientation, TR/TE = 4.6/2.1 ms, pulse duration = 1.86 ms, receiver bandwidth = 5405 Hz/pixel, mean FA = 4.70° and acquisition time = 19.1 s. Second, a FB variant of this scan (“FB 4.35 mm”) was performed with identical scan settings, except the acquisition time was 204 s. This led to R_uniq_ = 0.95 and R_tot_ = 0.10 (i.e., oversampling) before binning and R_uniq_ = 1.15 (i.e., almost full sampling) and R_tot_ = 0.38 for the expiration phase only. A value for R_uniq_ below 1 before binning was obtained because the spiral arms sampled slightly farther into the periphery of k‐space than a fully sampled acquisition with an elliptical shutter would. Third, a FB GRE was acquired with similar acquisition time (208 s) but a spatial resolution of 1.50 × 1.50 × 1.60 mm^3^ (“1.50 mm”), realized by using R_uniq_/R_tot_ = 1.49/0.73 (before binning) and R_uniq_/R_tot_ = 3.75/2.88 (expiration‐phase only). Other scan settings were FOV = 300 × 384 × 262 mm^3^, matrix size = 200 × 256 × 164, phase‐encoding directions RL and AP, coronal slice orientation, TR/TE = 4.6/2.1 ms, pulse duration = 1.86 ms and receiver bandwidth = 710 Hz/pixel. Fourth, a FB GRE was acquired with similar acquisition time (208 s) but a spatial resolution of 1.35 × 1.35 × 1.35 mm^3^ (“1.35 mm”), realized by using R_uniq_/R_tot_ = 1.72/0.96 (before binning) and R_uniq_/R_tot_ = 4.66/3.80 (expiration‐phase only). Other scan settings were FOV = 300 × 386 × 262 mm^3^, matrix size = 222 × 286 × 194, phase‐encoding directions RL and AP, coronal slice orientation, TR/TE = 4.6/2.1 ms, pulse duration = 1.78 ms and receiver bandwidth = 715 Hz/pixel. Finally, a FB GRE was acquired with roughly similar acquisition time (225 s) but a spatial resolution of 1.20 × 1.20 × 1.20 mm^3^ (“1.20 mm”). This was realized by using R_uniq_/R_tot_ = 1.42/0.67 (before binning) and R_uniq_/R_tot_ = 3.46/2.62 (expiration‐phase only), and a smaller FOV of 300 × 400 × 150 mm^3^, tailored to the participant's liver size in the AP‐dimension. Other scan settings were matrix size = 250 × 334 × 125, phase‐encoding directions RL and AP, coronal slice orientation, TR/TE = 4.6/2.1 ms, pulse duration = 1.86 ms and receiver bandwidth = 1837 Hz/pixel. All images were reconstructed without additional interpolation through k‐space zero filling.

Next, to show robustness, the 1.50 and 1.35 mm scans mentioned above were acquired for five more participants. For consistency, the same FOV with a large AP‐dimension as in Participant 1 was used for all participants, to ensure the abdomens would fit inside the FOV. For the 1.50 mm scan, TR = 4.6–4.7 ms, acquisition time = 205–212 s, pulse duration = 1.86–2.11 ms, and mean FA = 4.01–5.70°, varying between participants. For the 1.35 mm scan, TR = 4.6–4.7 ms, acquisition time = 208–212 s, pulse duration = 1.78–2.11 ms, mean FA = 4.01–5.70°, and receiver bandwidth = 715–832.8 Hz/pixel. Other scan settings were identical to those for Participant 1.

To provide a qualitative comparison between 3 T and 7 T liver MRI, we acquired additional scans at 3 T in two participants who were also included in the 7 T cohort (Participants 2 and 5). These scans were performed on an Ingenia 3 T scanner (Philips Healthcare, Best, The Netherlands) using the body coil in the scanner bore for transmission, and a 16‐channel anterior coil and 12‐channel posterior coil for reception. The 1.50 and 1.35 mm isotropic scans were acquired using the same sampling patterns, and hence acquisition times, as at 7 T. To accommodate for T_1_ differences and provide a fair comparison with 7 T, the flip angle and TE of the 3 T scans were varied. Firstly, for both participants, two variants of each scan were acquired. In Variant 1 (“FA1”), a lower flip angle (2.8°) was used to obtain equal liver T_1_‐weighting to the corresponding scan at 7 T, according to the spoiled gradient‐echo signal equation [28]. In Variant 2 (“FA2”), the Ernst flip angle (6.1°) was used. For both variants, liver T_1_ = 809 ms at 3 T was assumed [26]. Additionally, for the 3 T scan of Participant 2, a minimum TE of 1.45 ms was used to minimize T_2_*‐weighting. For Participant 5, a TE of 1.81 ms was used, leading to an equal level of water‐fat chemical shift effect of the 2^nd^ kind (“India ink artifact”) compared with the corresponding 7 T scan, assuming a water‐fat chemical shift of 3.5 ppm. The FOV, matrix size, phase‐encoding directions, slice orientation, TR and receiver bandwidth were matched to the corresponding 7 T scans, as listed above.

### 2.2 Image Reconstruction

FB GRE raw data were processed offline using ReconFrame (Gyrotools, Zurich, Switzerland) and BART [29]. To determine the respiratory signal, Fourier transformation and bandpass filtering were applied to the central k‐space lines (k_0_) in the FH‐direction, i.e., to the readout matrix with dimensions N_kx_ × N_ch_ × N_k0_, where N_ch_ is the number of receive channels. Subsequently, principal component analysis was employed to identify the components of respiratory motion. The outcome was a binned and subsampled k‐space with dimensions N_kx_ × N_ky_ × N_kz_ × N_ch_ × N_resp_ [30, 31].

For receive coil sensitivity map estimation, SAKE (simultaneous autocalibrating and k‐space estimation) [32, 33] was first employed to interpolate missing values near the center of k‐space that arose due to the pseudo‐spiral sampling and respiratory binning. The central 20 × 20 k‐space lines were filled using a 5 × 5 kernel with 50 fixed iterations. ESPIRiT [34] receive coil sensitivity maps were then estimated from this central k‐space data.

CS image reconstruction was conducted using BART's “PICS” command [29]. ℓ_1_‐regularization was performed using a Daubechies wavelet transform in the spatial dimensions, as described by the following equation:

(1) $$\underset{\text{m}}{\text{argmin}}\sum_{i}\left({\left\|y_{i}-F_{s}C_{i}m\right\|}_{2}^{2}\right)+\lambda {\left\|\mathrm{\psi m}\right\|}_{1}$$

where m is the reconstructed image, $F_{s}$ represents the undersampled Fourier transform, $C_{i}$ is the receive sensitivity map of channel *i*, y corresponds to the measured k‐space, ψ denotes a sparsifying transform, and λ controls the trade‐off between data consistency and sparsity via the wavelet transform. Data consistency is enforced using the squared ℓ_2_‐norm (i.e., the sum of squared differences between the measured and estimated k‐space), while signal sparsity is promoted by minimizing the ℓ_1_‐norm (i.e., the sum of the absolute values of the elements). This approach was exclusively applied to data acquired during the expiration phase, as B_0_ and phase shimming were performed on data acquired during breath‐holds at expiration. Finally, N4BiasFieldCorrection was applied to the reconstructed images to reduce residual B_1_^−^ inhomogeneity effects [35, 36]. Default parameter settings were chosen, with an isotropic spline spacing of 200 mm.

For 3 T, all reconstructions were performed similarly to 7 T, except no N4BiasFieldCorrection was applied. The regularization parameter was optimized individually for each scan, similarly to the 7 T reconstructions (see subsection Analysis), to ensure a fair comparison.

### 2.3 Analysis

To optimize the reconstruction pipeline and assess the effect of the number of respiratory bins on the quality of the reconstructed expiration‐phase images, the 1.50 and 1.35 mm scans were reconstructed using a varying number of bins (1, 2, 4, 6, 8, and 12) for Participant 2. Subsequently, for each reconstruction of the 1.50 and 1.35 mm scans of all participants, 25 values of the regularization parameter λ were evaluated, ranging from 10^−5^ to 10^−1^ on a logarithmic scale. Aside from qualitative assessment, the effect of varying bin number and λ on image sharpness was assessed quantitatively based on the maximum local variation (MLV) [37]. MLV captures the most significant variations in pixel intensities by calculating the maximum difference between a pixel intensity value ($I_{i,j}$) and those of its eight surrounding neighbors ($I_{x,y}$, with i−1≤x≤i+1 and j−1≤y≤j+1), i.e.:

(2) $$\mathrm{MLV}\left(I_{i,j}\right)=\max_{x,y}\left|I_{i,j}-I_{x,y}\right|$$

MLV was chosen over traditional methods such as the modulation transfer function (MTF) [38] or point spread function (PSF) [39] due to its robustness and simplicity in handling complex anatomical structures in MR images. Unlike MTF and PSF, which require specific assumptions about the imaging system and often necessitate ideal point or edge features for accurate measurement, MLV directly quantifies local intensity variations, making it more adaptable to the heterogeneous nature of liver tissue. Additionally, MLV can be computed efficiently across the entire image or a specific region of interest (ROI), providing a more comprehensive assessment of image sharpness without the need for extra preprocessing. MLV maps were calculated for a single‐slice ROI including the full liver and portal vein. Moreover, an image sharpness value was calculated from each of these MLV maps. The distribution of MLV values from every MLV map was parameterized by a generalized Gaussian distribution, including exponential weighting, according to Bahrami et al. [37]. The image sharpness value was then defined as the standard deviation of the resulting distribution, and was calculated using the method from Sharifi et al. [40]. Higher MLV values across an ROI therefore correspond to higher sharpness values. Sharpness values were normalized against the maximum value across the parameter search, for both 1.50 and 1.35 mm.

## 3 Results

**FIGURE 2:**
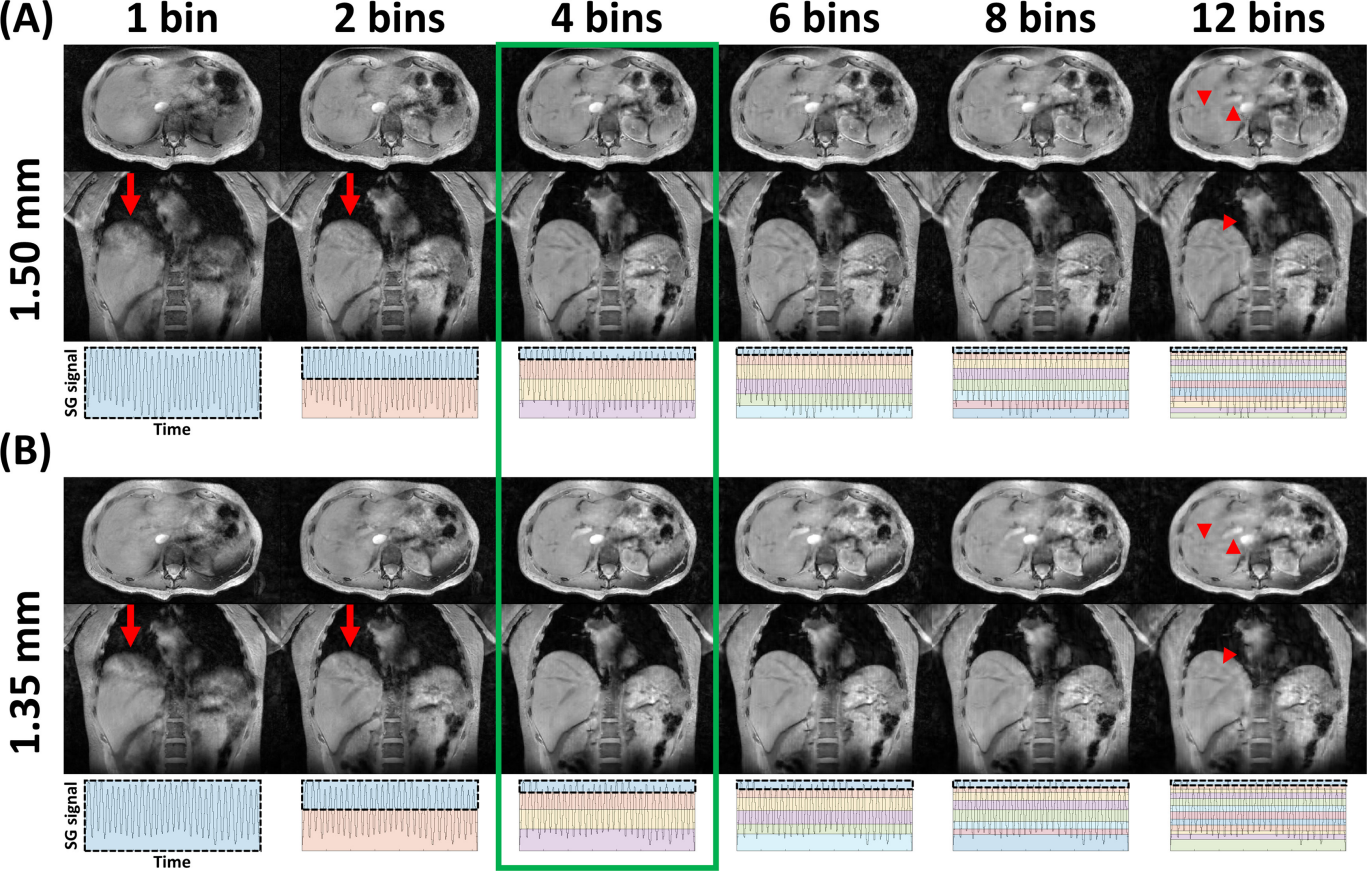
Expiration‐phase axial (top row) and coronal (middle row) images from the 1.50 mm (A) and 1.35 mm (B) acquisitions, reconstructed using a varying number of respiration bins, in Participant 2. Image reconstruction was performed using regularization factors of 10^−2.5^ for 1.50 mm and 10^−2.33^ for 1.35 mm (see also Figures 3 and 4). For each number of bins, images were reconstructed from the subset of data in the top bin (i.e., containing the peaks of the self‐gating signal (bottom row, black dotted box)). Red arrows highlight residual motion artifacts due to low bin number, whereas red arrowheads highlight loss of detail due to a high undersampling factor. The green rectangle highlights the selected optimal number of bins. SG: self‐gating.

Figure 2 shows expiration‐phase images from the 1.50 and 1.35 mm acquisitions, reconstructed using a varying number of respiration bins, for Participant 2. Observations were comparable between 1.50 and 1.35 mm. When a low number of bins was used (1 and 2), residual motion artifacts were visible near the liver dome (red arrows). Conversely, using a high number of bins (6, 8, and 12) removed motion artifacts, resulting in a sharply delineated liver dome. However, the high undersampling factor of the expiration‐phase dataset led to loss of detail in several structures, with increasing artifact severity for higher bin numbers (red arrowheads for the example of 12 bins). From a visual assessment, four bins were selected as the number qualitatively optimizing the tradeoff between these two artifact types, giving the highest image quality (green rectangle).

**FIGURE 3:**
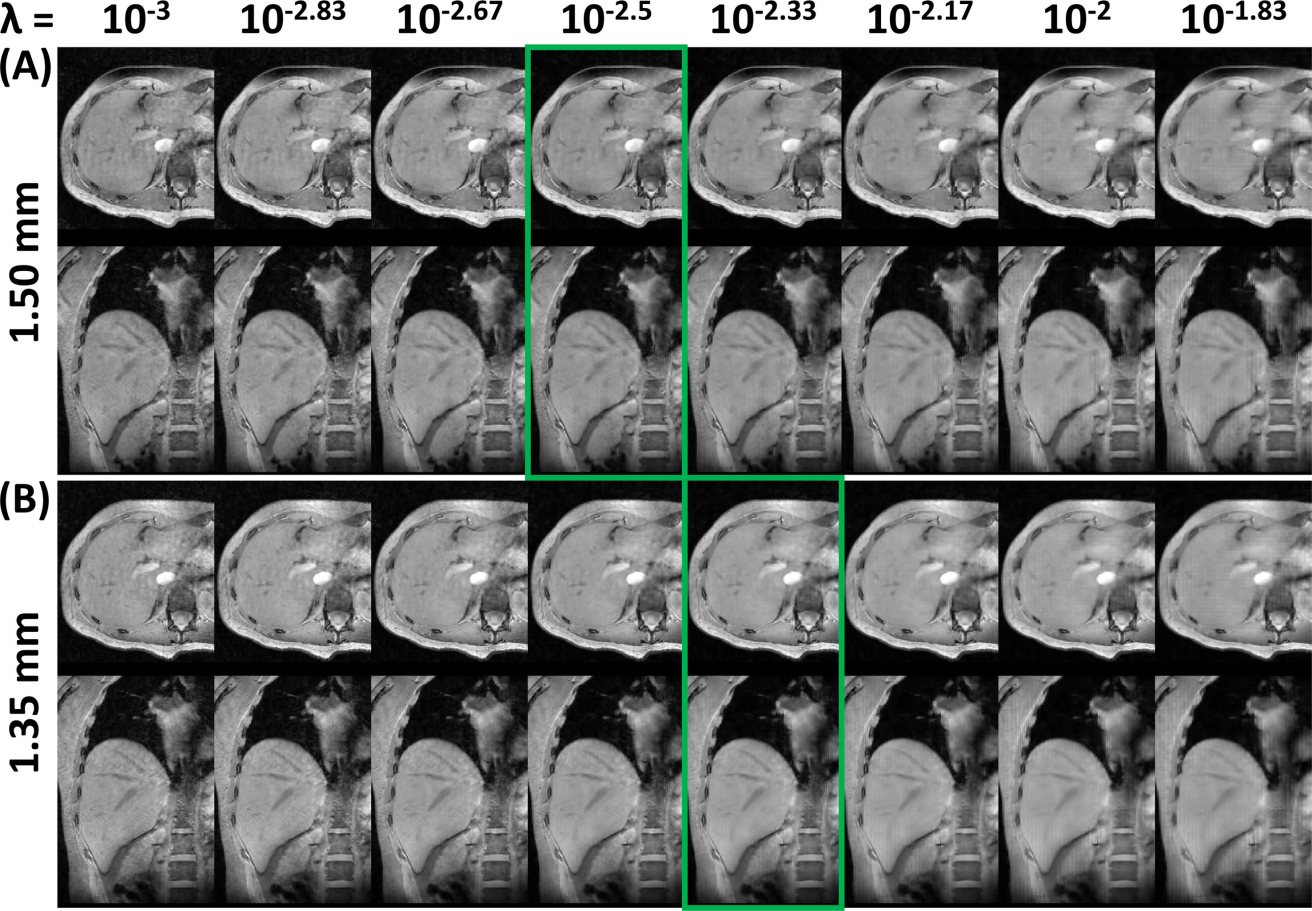
Cropped axial (top row) and coronal (bottom row) images from 1.50 mm (A) and 1.35 mm (B) acquisitions, reconstructed using a varying ℓ_1_‐regularization factor (λ), for the same participant and slice as in Figure 2. Image reconstruction was performed using four bins (see also Figures 2 and 4). To retain clarity, only eight of the 25 tested values for λ are shown. The green rectangles highlight the selected optimal values for λ.

Figure 3 shows cropped images from the 1.50 and 1.35 mm acquisitions, reconstructed using a varying ℓ_1_‐regularization factor, for the same slice of the same participant as in Figure 2. The regularization factor λ determines the tradeoff between increased SNR (high λ) and increased data consistency (low λ), characteristic of CS reconstruction, which can be appreciated in both orientations at both 1.50 and 1.35 mm. Based on visual inspection, values for λ of 10^−2.5^ (1.50 mm) and 10^−2.33^ (1.35 mm) were selected as qualitatively optimizing this tradeoff (green rectangles). The higher optimized value of λ for higher‐resolution data is likely to be a result of the lower SNR at 1.35 mm resolution than at 1.50 mm resolution.

**FIGURE 4:**
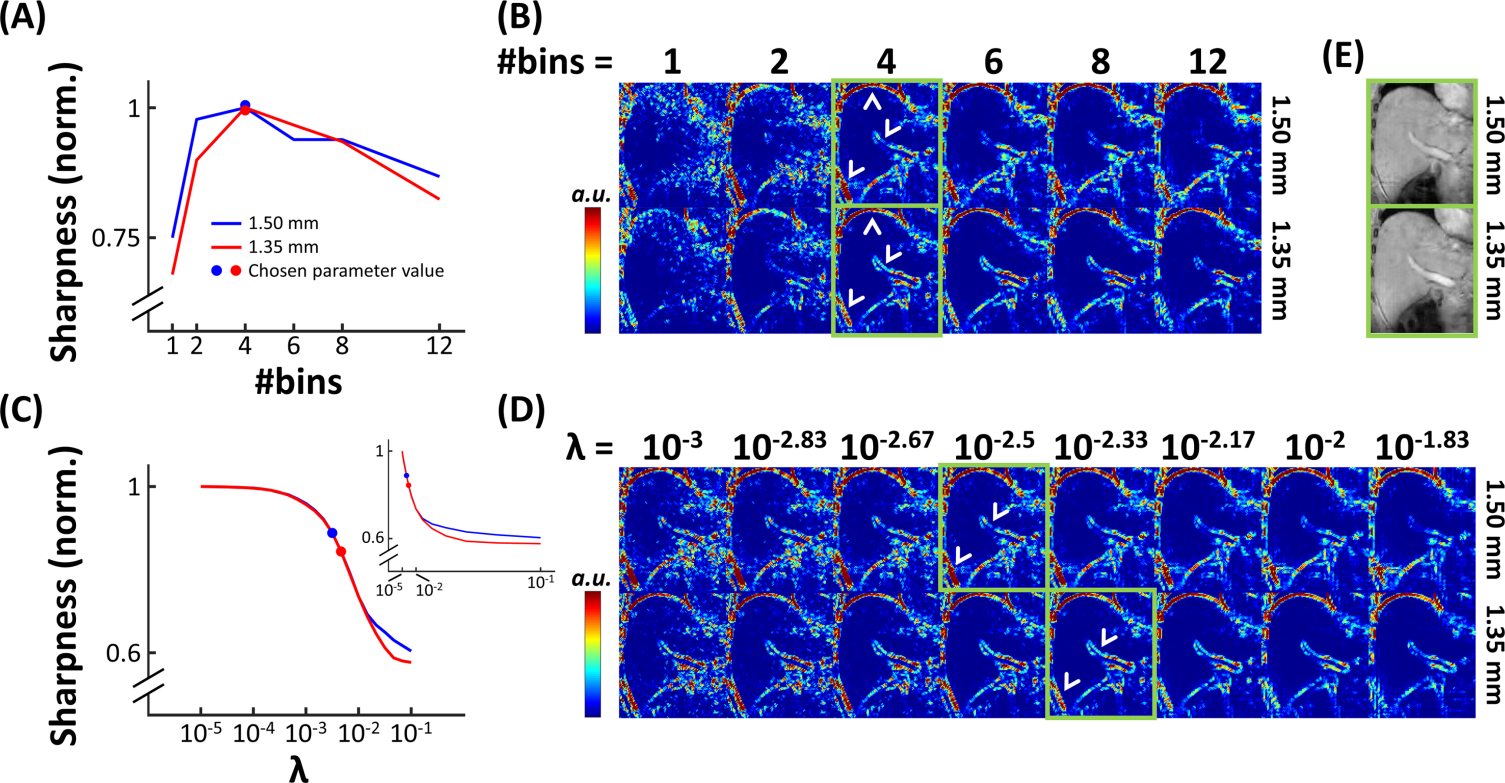
Quantitative assessment of sharpness (A, C) and MLV maps (arbitrary unit, B,D) of the 1.50 and 1.35 mm acquisitions from a single representative participant (i.e., the same participant as in Figures 2 and 3), evaluated for a single‐slice ROI (E). (A, C) Plots of sharpness vs. bin number and λ for 1.50 mm (blue) and 1.35 mm (red). To retain clarity, the sharpness and MLV map values for varying bin number (A,B) were only shown using λ = 10^−2.5^ (1.50 mm) and λ = 10^−2.33^ (1.35 mm, see Figure 3), and those for varying λ (C, D) were only shown using four bins (see Figure 2). Sharpness values were normalized against the maximum value across the parameter search, for both 1.50 mm and 1.35 mm. In (C), the insert image shows the same plot using a linear scale for λ. The green rectangles (B, D) and red and blue circles (A, C) highlight the selected optimal values for the bin number and λ. The images in (E) were reconstructed using these parameters. White arrowheads (B,D) highlight areas with high MLV when using these parameters.

The quantitative assessment of sharpness and MLV maps is shown in Figure 4. In the plot with varying bin number from a single representative participant (Participant 2; Figure 4A), sharpness was highest when using four bins for both 1.50 and 1.35 mm. This can also be appreciated in the MLV maps in Figure 4B. For low bin number (1 and 2), sharpness of the liver boundary and portal vein was lost. For higher bin numbers, MLV values at these locations increased, indicating higher sharpness (white arrowheads). However, for too high bin numbers, although the liver dome remained clearly delineated, sharpness of other structures was lost again due to a high undersampling factor. In the plot with varying λ (Figure 4C), a monotonic decrease in sharpness was seen for increasing λ for both 1.50 and 1.35 mm. This is intuitive, considering the enhanced denoising effect for increased regularization and the fact that noise leads to large spatial variations and thus high MLV values. Likewise, the MLV maps in Figure 4D showed the highest values for low λ. For higher λ, high MLV values from noisy pixels started to decrease, but artifacts and loss of detail (for example at the portal vein) arose for too high λ. Upon visual review, values for λ of 10^−2.5^ (1.50 mm) and 10^−2.33^ (1.35 mm) were selected as providing maximum denoising without suffering from overregularization (green rectangles). In the sharpness curve of Figure 4C, this corresponded to the beginning of the sharp decline (blue and red circles). These results confirm the qualitative findings from Figures 2 and 3. Therefore, reconstructions of all other participants were also performed using four bins. Although λ was optimized separately for each participant, findings were comparable to the participant used in Figures 2, 3, 4, and the optimal values λ = 10^−2.5^ (1.50 mm) and λ = 10^−2.33^ (1.35 mm) were used for all 7 T participants. Overall, no clear increase or loss in sharpness was observed for 1.35 mm compared to 1.50 mm.

**FIGURE 5:**
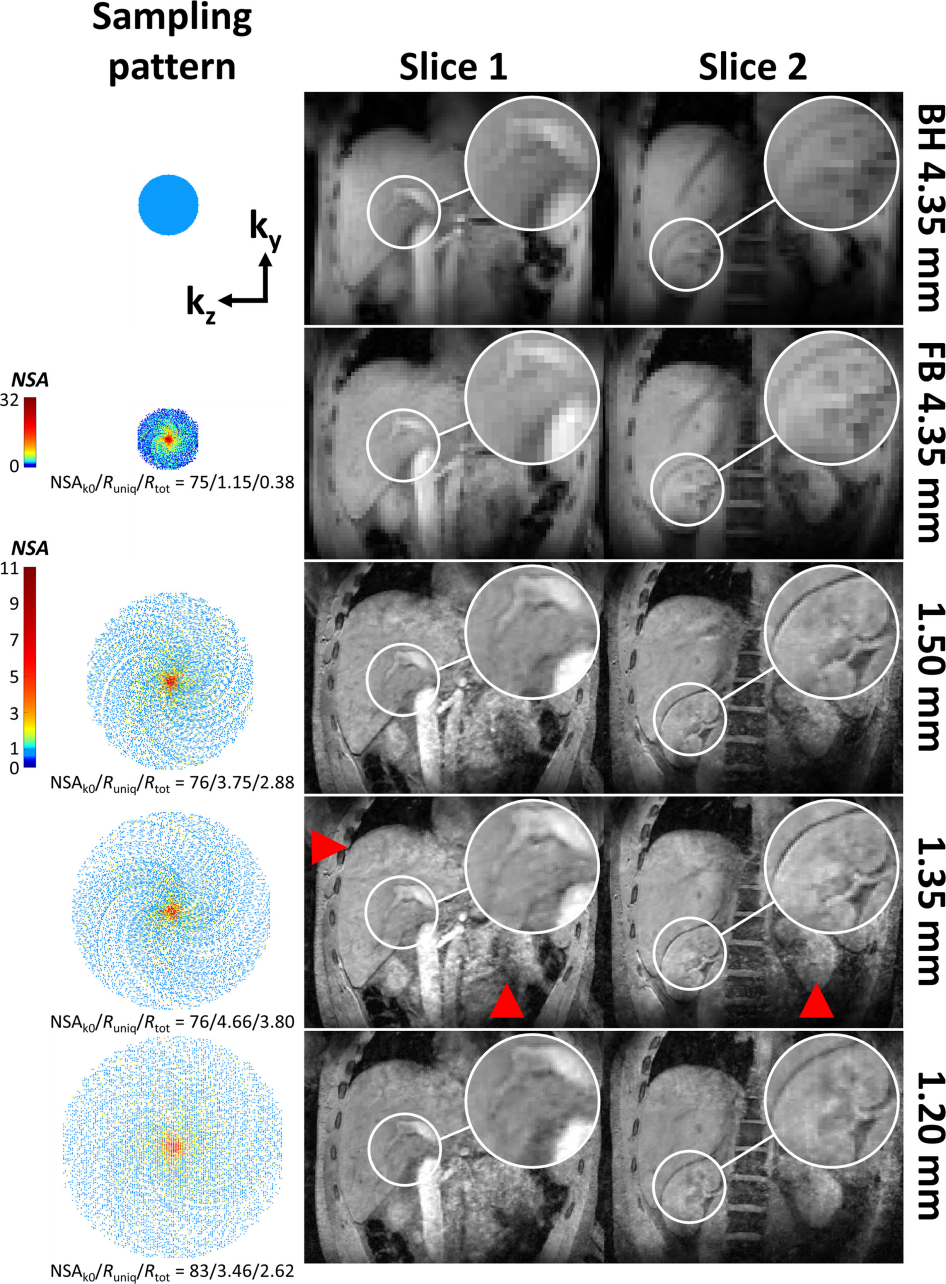
K‐space sampling patterns (left) and two coronal slices (right) from five acquisitions with increasing matrix size in Participant 1. In the sampling patterns, each point represents a fully sampled k_x_ readout line. Note that the larger k‐point spacing in the k_z_‐direction for 1.20 mm reflects the FOV‐reduction in the AP‐direction. For the variable‐density averaging patterns of 1.50, 1.35, and 1.20 mm, a different color scale than for FB 4.35 mm was used, because of the lower NSA values close to the k‐space center. Red arrowheads highlight artifacts in the high‐resolution scans, for the example of 1.35 mm. BH: breath‐hold. FB: free‐breathing. NSA_k0_: number of signal averages of the k‐space center.

Figure 5 shows the k‐space sampling patterns and two coronal slices from the five scans with increasing matrix size for Participant 1. The breath‐hold scan (“BH 4.35 mm”, first row) showed a pixelated appearance due to its small matrix size. The translation of this scan to a FB acquisition with a negligible undersampling factor (“FB 4.35 mm”, second row, R_uniq_ = 1.15) showed no loss in image quality and comparable image sharpness. Although FB 4.35 mm contained a factor of 1/0.38 = 2.63 more sampled k‐space points compared to BH 4.35 mm, no increase in perceived SNR was observed. B_1_^+^ inhomogeneity‐related signal variations could be removed successfully using phase shimming in both the BH and FB acquisitions. This resulted in a homogeneous appearance across the entire liver, even including structures outside the VOI such as the inferior vena cava (IVC, Slice 1), the right kidney and the vertebrae (Slice 2). Next, extending this fully sampled FB scan to an undersampled acquisition with similar acquisition time but increased matrix size (“1.50 mm”, third row, R_uniq_ = 3.75), showed a substantial increase in image sharpness while maintaining perceived SNR. The two coronal slices highlight the clearly increased detail of the IVC and portal vein (Slice 1), and the liver‐kidney boundary and renal structures (Slice 2). Increasing the resolution to 1.35 mm (“1.35 mm”, fourth row) and 1.20 mm (“1.20 mm”, fifth row) isotropic, with maintained acquisition time, did not show an observable increase in image sharpness. The 1.35 mm scan showed comparable image sharpness and quality compared to 1.50 mm, while 1.20 mm showed a slight decrease in sharpness compared to 1.50 and 1.35 mm, despite R_uniq_ and R_tot_ of 1.20 and 1.50 mm being comparable. Contrary to BH 4.35 mm and FB 4.35 mm, the high‐resolution scans showed residual artifacts that are particularly prominent near the liver dome and to the contralateral side of the liver (red arrowheads for the example of 1.35 mm).

**FIGURE 6:**
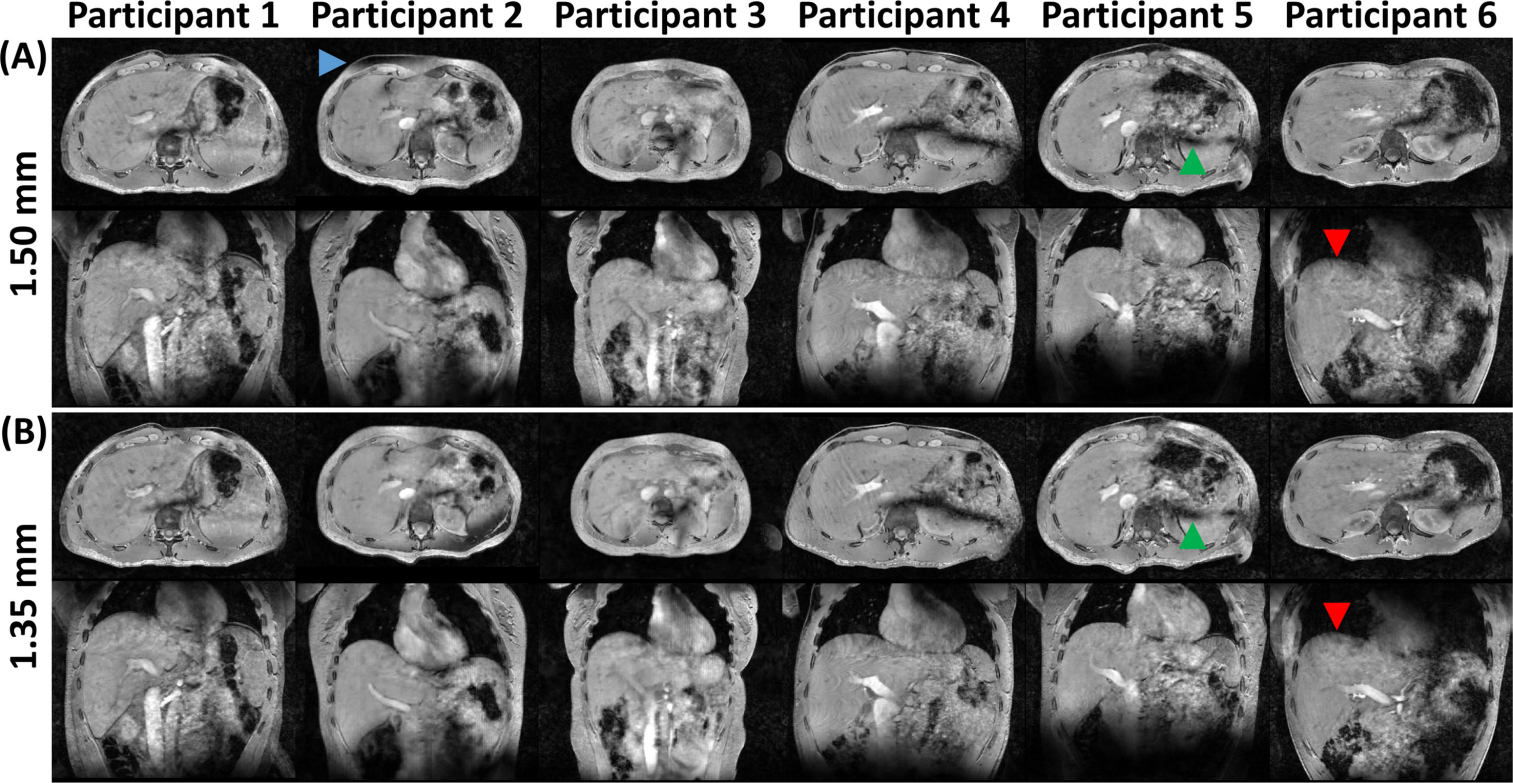
Axial (top row) and coronal (bottom row) images from the 1.50 mm (A) and 1.35 mm (B) acquisitions in all six participants. Red arrowheads highlight blurring near the liver dome, and the blue arrowhead highlights a loss of signal in subcutaneous fat. Green arrowheads highlight areas of B_1_^+^ inhomogeneity outside of the liver.

Figure 6 shows images from the 1.50 and 1.35 mm acquisitions in all six participants. The findings from Participant 1 in Figure 5 translated to all participants. Images with high isotropic resolution and perceived SNR were obtained for all participants, leading to detailed visibility of anatomical structures. Moreover, the use of phase shimming consistently eliminated B_1_^+^ inhomogeneity effects across the entire liver, moving them outside of the VOI (see green arrowheads for the example of Participant 5). Like in Figures 2, 3, 4, 5, no clear increase or loss in detail was observed for 1.35 mm compared to 1.50 mm. Varying levels of artifact severity were observed for different participants. For example, for both 1.50 and 1.35 mm, Participants 1, 3, and 6 showed blurring near the liver dome, compared to a sharp delineation for Participants 2, 4, and 5 (red arrowheads for the example of Participant 6). For several participants, a loss of signal in subcutaneous fat (not included in the VOI) was observed, which is probably related to B_0_‐inhomogeneity (blue arrowhead for the example of Participant 2).

**FIGURE 7:**
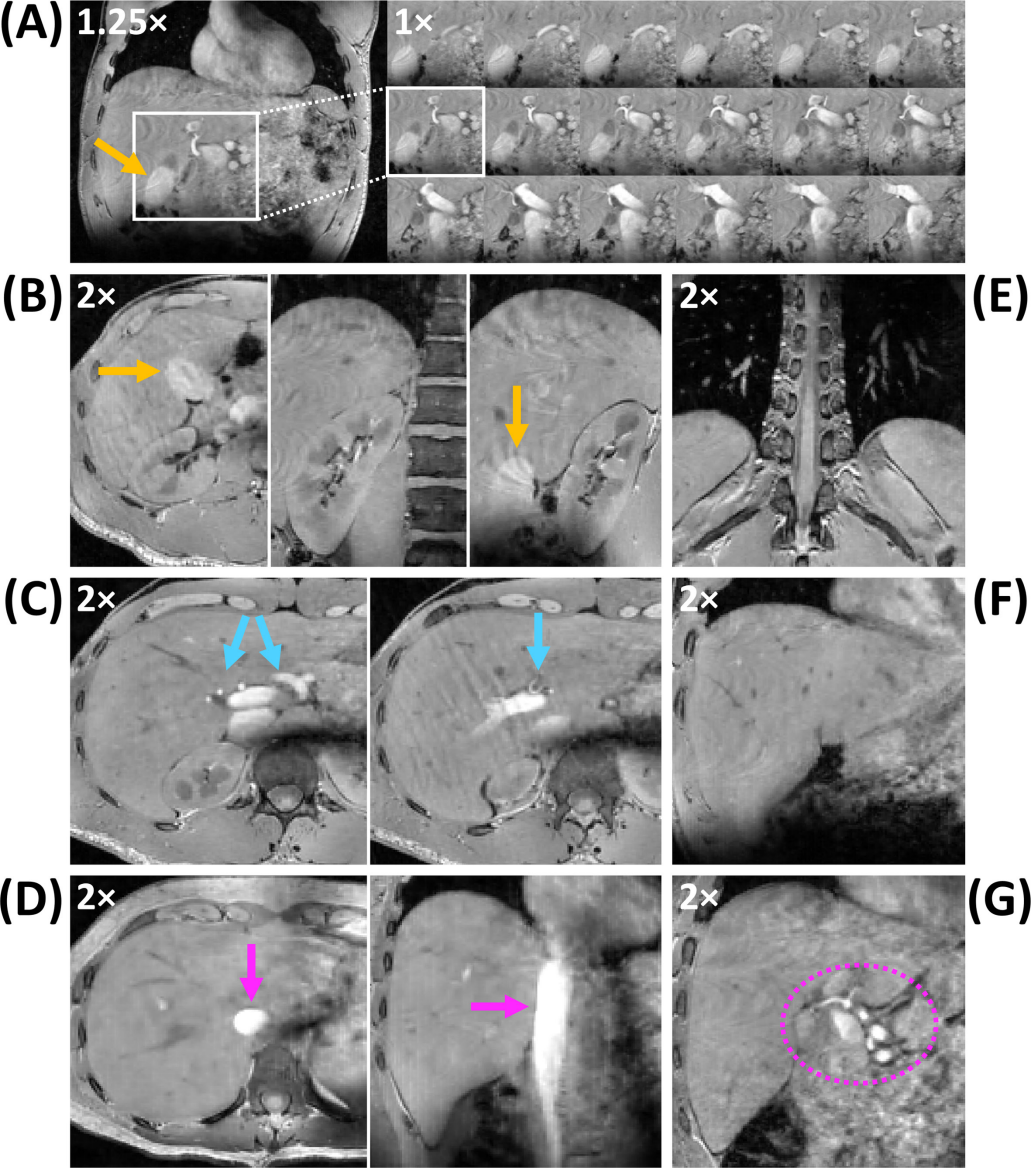
Visualization of multiple structures on the 1.35 mm scans of different participants. (A) Cropped image of 18 coronal slices of Participant 4 showing the hepatic artery, portal vein and IVC. (B) Three orthogonal views of Participant 4 centered on the kidney. The renal cortex, medulla, and vasculature are visible, as are the vertebrae. The gall bladder is highlighted by orange arrows (A, B). (C) Two axial slices of Participant 4 showing the portal vein and hepatic arteries (blue arrows). (D) Axial and coronal slice of Participant 2 showing the IVC (purple arrows). (E) Coronal slice of Participant 4 showing the spinal cord, vertebrae, and pulmonary vessels in both lungs. (F) Coronal slice of Participant 4 showing hepatic vessels and bile ducts. (G) Coronal slice of Participant 1 showing the hepatic artery and portal vein (purple dotted circle). Zoom factors are shown in the top left for each subfigure.

Figure 7 shows the clear depiction of multiple structures on the 1.35 mm scans of different participants, including the kidney, gall bladder, and several blood vessels. Despite the lack of contrast agent, a hyperintense signal was observed in multiple blood vessels for all participants. Figure S1 shows a video cycling through coronal slices of the 1.35 mm acquisition in Participant 2, highlighting the high three‐dimensional isotropic resolution that can be obtained throughout the entire liver using the proposed method.

**FIGURE 8:**
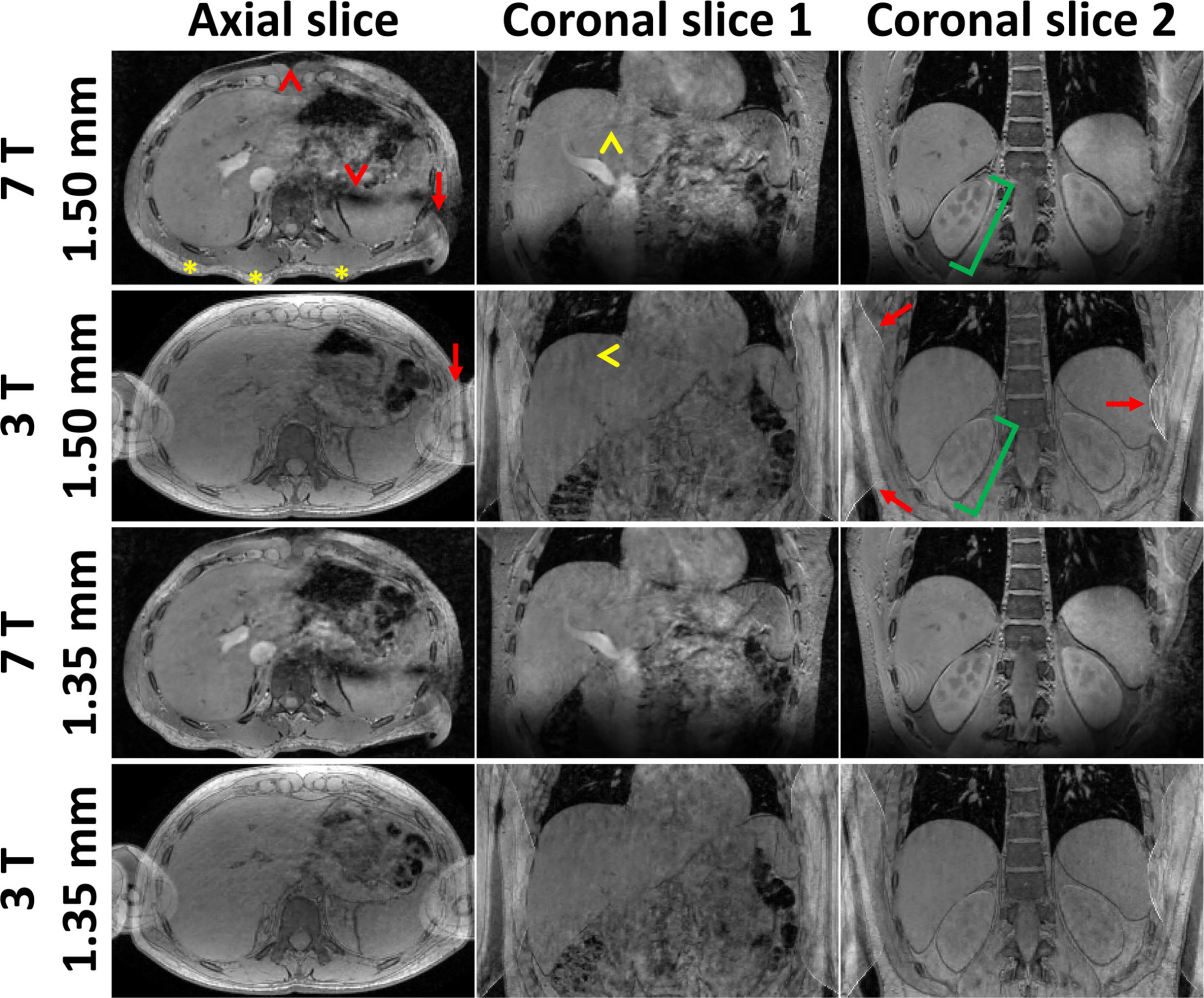
Comparison of 3 T and 7 T results for Participant 5, showing one axial and two coronal slices at isotropic resolutions of 1.50 and 1.35 mm. The 3 T scans were acquired using a flip angle (FA1) that provided equivalent liver T_1_‐weighting to 7 T. Green brackets highlight increased renal corticomedullary contrast at 7 T compared to 3 T. Yellow arrowheads highlight “wavy” artifacts throughout the liver for both field strengths, and red arrows highlight fold‐over artifacts. Red arrowheads highlight signal dropouts in the subcutaneous fat (B_0_‐related) and to the contralateral side of the liver (B_1_^+^‐related) for 7 T, which are no issue at 3 T. The posterior bumps for 7 T were caused by the rectangular dipoles in the transmit‐receive coil (yellow asterisks). For clarity, annotations were omitted for the 1.35 mm scans.

**FIGURE 9:**
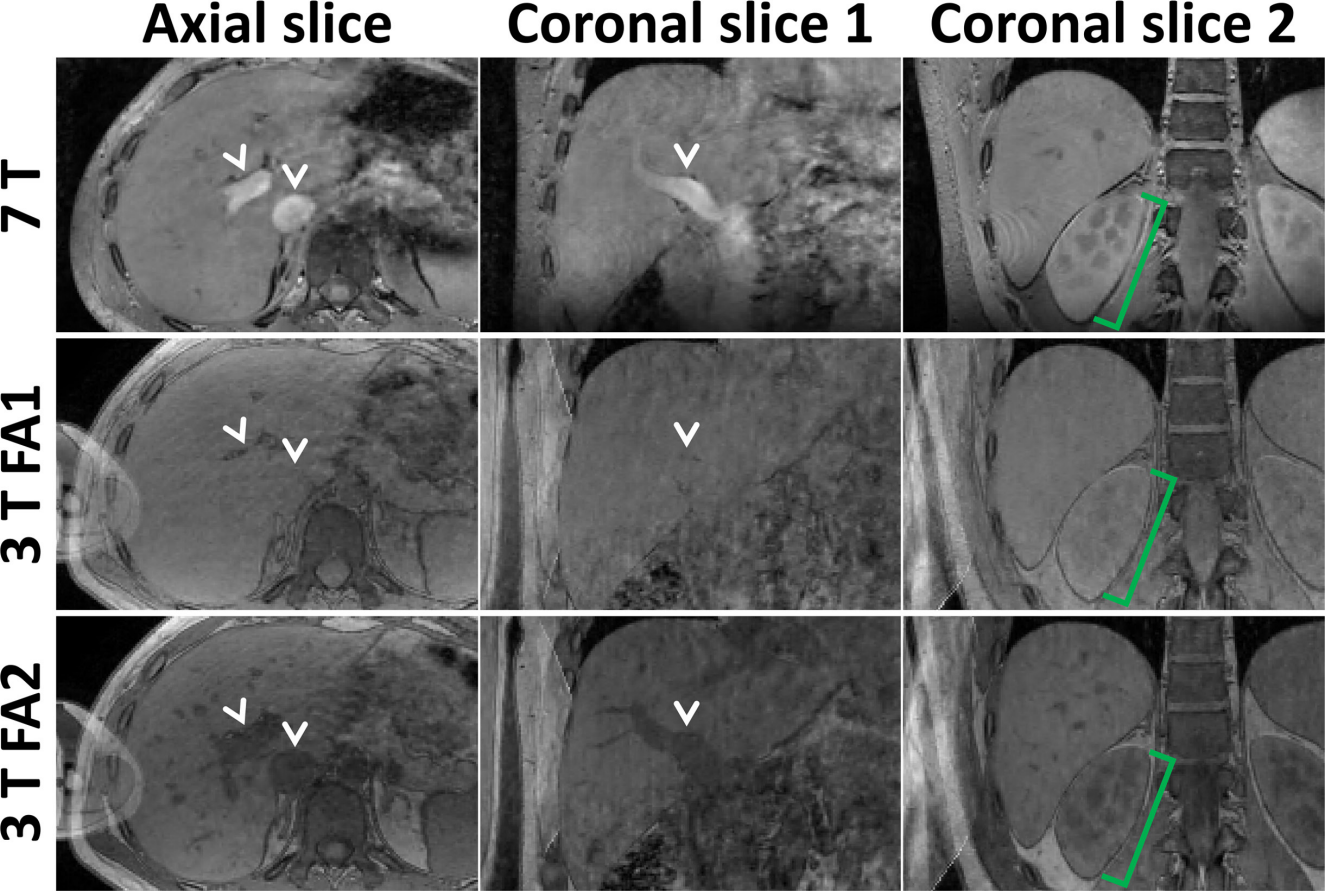
Comparison of 7 T results (using Ernst angle) with the 3 T results acquired using low flip angle (FA1, leading to equal liver T_1_‐weighting to 7 T) and Ernst angle (FA2), for 1.35 mm resolution in Participant 5 (1.5× zoomed‐in with respect to Figure 8). White arrowheads highlight vasculature, appearing bright at 7 T, indistinguishable for 3 T FA1 and dark for 3 T FA2. Green brackets highlight increased renal corticomedullary contrast at 7 T compared to 3 T (FA1 and FA2).

Figure 8 compares 3 T and 7 T results from Participant 5, showing one axial and two coronal slices at isotropic resolutions of 1.50 and 1.35 mm, using a flip angle that equalized liver T_1_‐weighting across field strengths (FA1). Figure S2 shows the same for Participant 2. Overall, image sharpness and SNR appeared comparable between 3 T and 7 T. As was already observed in Figure 6 for 7 T, a “wavy” artifact throughout the liver was observed for both field strengths in Participant 5 (yellow arrowheads), which was less apparent in Participant 2. Interestingly, the hyperintensity in blood vessels observed at 7 T was not observed at 3 T, leading to indistinguishable liver‐vessel signal (see also Figures 9 and S3). For both participants and resolutions, renal corticomedullary contrast was more pronounced at 7 T than at 3 T (green brackets in Figure 8). Another notable difference was the presence of fold‐over artifacts at 3 T, which were much less severe at 7 T (red arrows), due to the different coil architecture. The higher TE for the 3 T‐scan of Participant 5 led to a more similar appearance between field strengths near water‐fat boundaries, compared to Participant 2 (blue arrowheads in Figure S2). No noticeable further differences due to different TE between 3 T participants and field strengths were observed, e.g., in terms of T_2_*‐weighting. Similar to 7 T, no clear gain or loss in sharpness was observed between 1.50 and 1.35 mm at 3 T.

Figure 9 compares the 7 T results (using Ernst angle) with the 3 T results acquired using low flip angle (FA1, leading to equal liver T_1_‐weighting to 7 T) and Ernst angle (FA2), for 1.35 mm resolution in Participant 5. Figure S3 shows the same for Participant 2. Compared to FA1, usage of the Ernst angle (FA2) at 3 T did not lead to an observable SNR increase in the liver, again resulting in comparable image sharpness and perceived SNR between field strengths. Contrast dramatically increased for FA2 for multiple structures, leading to a more interesting appearance throughout the liver and right kidney. Still, even for FA2, renal corticomedullary contrast was more pronounced at 7 T than at 3 T for both participants (green brackets in Figure 9). While using the Ernst angle at 7 T led to hyperintense blood vessels, they appeared dark at 3 T (white arrowheads). Findings were comparable between both participants (Figures 9 and S3).

## 4 Discussion

In this work, a FB, high‐resolution, CS‐accelerated 3D GRE sequence was proposed for 7 T liver imaging. First, it was shown that using respiratory binning based on self‐gating, end‐expiration‐phase images with maintained image quality can be obtained from near fully sampled FB acquisitions compared to a breath‐hold scan of the same resolution (4.35 mm). Second, we showed that using golden‐angle pseudo‐spiral undersampling with CS reconstruction, a spatial resolution of up to 1.35 mm isotropic can be obtained in the same scan time (204–208 s), providing clear visualization of liver anatomy. High signal homogeneity could be achieved in all 13 acquisitions (6 × 1.50 mm, 6 × 1.35 mm, 1 × 1.20 mm), using eight‐channel phase shimming based on expiratory‐phase Fourier PE‐DREAM B_1_^+^ mapping and N4BiasFieldCorrection, i.e., without complex pulse design such as spokes [19, 41] or *k*_T_‐points [20, 42].

Contrary to the near fully sampled FB acquisition with 4.35 mm resolution, artifacts started to appear in the undersampled high‐resolution scans, particularly near the liver dome. Therefore, with the current eight receive channels and regularization only in the spatial domain, using lower undersampling factors could offer improvement. Also, increasing spatial resolution slightly from 1.50 to 1.35 mm isotropic did not result in sharper images with our current approach. Interestingly, increasing resolution further to 1.20 mm in the same scan time led to decreased rather than increased image sharpness for the single participant investigated, despite the undersampling factor being lower than for both 1.50 and 1.35 mm. We hypothesize that the more rectangular k_y_‐k_z_ matrix size for 1.20 mm, due to the reduced FOV in the AP‐direction compared to 1.50 and 1.35 mm, might lead to less favorable conditions for CS reconstruction, and that further optimization of the undersampling pattern might offer improvement. However, reduction in SNR due to voxel shrinkage could also play a role.

The MRI sequence used here is a building block in multiple clinical liver MRI applications involving GREs [43], such as fat fraction [44] and iron deposition [45] quantification, and 4D Flow [46]. In particular, low‐flip‐angle T_1_‐weighted spoiled 3D GRE sequences have been the cornerstone for the detection and characterization of liver lesions in DCE MRI at clinical field strengths [47]. DCE liver MRI involves a challenging tradeoff between optimizing volumetric coverage, spatial resolution and temporal resolution, especially considering the narrow time window of the arterial and portal venous phases [43]. For example, Pandharipande et al. advocate that in volumetric liver perfusion MRI, a minimal isotropic spatial resolution of 2 mm and temporal resolution of 3 s should be aimed for [48]. Therefore, many studies have employed undersampling techniques, either with parallel imaging with or without CS [49, 50, 51, 52, 53] or deep learning reconstructions [54] to obtain high spatial resolution within breath‐hold acquisition times. However, such relatively long breath‐holds reduce patient comfort, and motion artifacts can result in patients having difficulty holding their breath repeatedly, for example in elderly or pediatric cohorts. Consequently, much work has been done to extend those acceleration techniques to a FB framework, often using XD‐GRASP [8, 55, 56, 57, 58, 59] or similar [60]. For example, Chandarana et al. performed XD‐GRASP DCE at 1.5 T with respiratory binning, using a CS reconstruction, which exploited sparsity along both the contrast‐enhancement and respiratory‐phase temporal dimensions [55]. This resulted in end‐expiratory‐phase XD‐GRASP reconstructions with spatial and temporal resolutions of 1.5 × 1.5 × 3 mm^3^ in 14 s, respectively, which outperformed conventional breath‐hold acquisitions [55]. Recently, Chen et al. used XD‐GRASP at 3 T for contrast‐enhanced imaging of the hepatobiliary phase, for which the constraint on the temporal resolution is much less severe [43]. They obtained end‐expiratory‐phase images with spatial resolution of 1.03 × 1.03 × 4.10 mm^3^ (before interpolation) in 138 s, which showed significantly higher liver edge sharpness, hepatic vessel clarity, and lesion conspicuity compared with a breath‐hold counterpart [59]. In this study, we performed a similar experiment at 7 T, except with pseudo‐spiral Cartesian instead of radial stack‐of‐stars sampling, and isotropic voxel sizes (allowing for multiplanar reconstructions). The pseudo‐spiral sampling pattern combines the advantages of both Cartesian and non‐Cartesian acquisitions. It offers non‐Cartesian benefits, such as improved sampling efficiency and reduced motion artifacts, while also allowing for simple reconstruction using fast Fourier transforms, similar to Cartesian data. In addition, the pseudo‐spiral sampling pattern addresses the problem of variations in the quality of CS reconstructed images, which can often arise due to the random undersampling patterns required in traditional CS with Cartesian acquisition [9, 61, 62]. By adopting the pseudo‐spiral approach, we can achieve a more uniform and predictable sampling density, leading to more consistent and higher‐quality image reconstruction.

Our voxel volume ((1.35 mm)^3^ = 2.46 μL) was smaller than that of Chen et al. (4.36 μL), with a relatively short scan time (208 vs. 138 s) [59]. Therefore, hepatobiliary‐phase DCE could be a suitable application for our proposed technique, especially if scan time is reduced further by using a participant‐tailored FOV in the AP‐direction, like in the 1.20 mm acquisitions. Conversely, our scans would have to get much shorter to allow adequate multiphase DCE MRI. Hence, in the future we intend to increase the undersampling factor by exploiting sparsity in one or two temporal dimensions, similarly to Chandarana et al. [55]. Further application‐specific sequence optimization might also be beneficial, such as using higher flip angles for increased T_1_‐weighting and adding fat suppression, which is relevant in liver MRI. Moreover, if a large enough FOV is chosen that covers the liver dome to allow for self‐gating, it could also be used in other, smaller abdominal organs of interest, such as the pancreas, prostate, or kidneys. Lastly, the application of our technique in other sequences such as T_2_‐weighted or diffusion MRI could be explored, in which the use of larger flip angles could be more challenging, especially when multiple refocusing pulses are employed.

The main obstacle hindering us from exploiting sparsity in the respiratory temporal dimension was that our phase‐shimmed pulses were optimized for expiration‐phase B_0_ and B_1_^+^ maps only. For 7 T cardiac MRI, Aigner et al. [63] showed that pulses tailored to a specific respiration phase do not necessarily produce homogeneous images in other phases, which they solved by calculating respiration‐robust pulses from respiration‐resolved B_1_^+^ maps [64]. Although our approach would benefit from such respiration‐robust pulses, the impact of respiratory motion on B_1_^+^ inhomogeneity might be less severe for our liver acquisitions. Figure S4 displays coronal slices from four respiratory phases (end‐inspiration to end‐expiration) for Participant 2 and Figure S5 shows the same in a video. Aside from other artifacts in the liver, for this participant we found that signal dropouts that were moved outside the liver due to phase shimming, remained outside the liver during the whole respiration cycle (green dotted ellipses). Therefore, future work should show to what extent our phase‐shimmed pulses have to be altered to allow for high‐quality liver images along the whole respiratory cycle. This would not only allow regularization along the respiratory dimension, which could increase image quality, but also potentially increase efficiency by combining data from multiple respiration phases using registration techniques [30, 65]. Alternatively, information from multiple respiration phases may also have relevance in multiple clinical problems [8]. Lastly, Aigner et al. also showed that pTx pulses can be made universal (calibration‐free) [66, 67], which would be highly beneficial for our approach, which currently requires 13 breath‐holds of 12.6 s for B_1_^+^ mapping.

Using four respiration bins during image reconstruction led to the highest level of image quality for the investigated participant, in agreement with other studies [8, 30]. Due to the high computational cost associated with evaluating six different bin numbers and 25 different values for λ, resulting in 150 reconstructions per participant, binning was optimized only for Participant 2 rather than individually for each participant, which is common practice in the literature [7, 68]. For several participants, residual blurring was observed near the liver dome in coronal images, which could be motion‐related. Therefore, it is conceivable that the optimal number of respiration bins varies on a case‐by‐case basis, and that case‐specific optimization may provide additional improvements. This holds in particular if data from respiratory phases other than end‐expiration are to be used. Figures S4 and S5 show that whereas a sharp liver dome delineation was obtained for end‐expiration, residual blurring was observed for the other three respiratory phases, possibly motivating a varying number of bins or bin sizes for such an application. These figures do show that the respiration cycle was captured accurately, and that the temporal k‐space center sampling frequency of 1/690 ms^−1^ for self‐gating was sufficient.

The BMI values of the participants included in this study were in the normal range of 18.5–24.9 kg/m^2^ and only one participant had a relatively high body circumference (97 cm) [69]. Therefore, it remains to be investigated whether re‐optimization of the number of bins, as described above, is required in obese participants. Employment of pTx with Fourier PE‐DREAM B_1_^+^ mapping, on the other hand, is expected to be feasible, based on results in a previous paper employing the same technique in an obese participant [20].

Aside from B_1_^+^ inhomogeneity, increased susceptibility effects are another challenging aspect of UHF liver MRI, especially when GREs are employed. Susceptibility artifacts are known to be particularly severe at air‐tissue interfaces, such as between the lung and liver. This could be another explanation why artifacts were more prevalent near the liver dome in this study. It could also explain why the non‐expiration‐phase images in Figures S4 and S5 show more severe artifacts, as they are reconstructed using data from a larger range of motion states, i.e., more mixing of lung and liver voxels. This effect is exacerbated further because B_0_ inhomogeneity itself is expected to be larger at higher field strengths, and additionally, B_0_ field fluctuations are expected due to breathing. Therefore, just as was alluded to for B_1_^+^ shimming earlier, implementing respiration‐robust, real‐time, and/or higher‐order B_0_ shimming might offer further improvement.

UHF MRI is also known to suffer more from B_1_^−^‐related inhomogeneity, due to the lack of a homogeneous reference body coil image typically used in correction methods [70]. Retrospective application of N4BiasFieldCorrection largely mitigated this problem in our study, leading to images with high degree of homogeneity for all acquisitions. Optimizing N4BiasFieldCorrection parameter settings on a case‐by‐case basis could be preferred to improve this further. However, more advanced strategies might be considered, such as uniform combined reconstruction [71] or incorporating bias correction into the PICS reconstruction [72], which could lead to even more clinically attractive images.

At 7 T, a handful of liver MRI feasibility trials have been performed, in which T_1_‐weighted 3D GRE scans were acquired in a single breath‐hold using parallel imaging with a factor of 2 [3, 73, 74, 75]. Umutlu et al. [73] (voxel size: 1.25 × 1.25 × 1.6 mm^3^) and Fischer et al. [75] (1.25 × 1.25 × 1 mm^3^) demonstrated the potential of multi‐phase DCE in the liver vasculature and biliary tract (MR cholangiography), respectively. Laader et al. [3] (0.8 × 0.8 × 3.0 mm^3^) reported that arterial vasculature delineation was rated significantly higher at 7 T compared to 1.5 T and 3 T, leading to high‐quality conspicuity of intra‐abdominal vessels, despite the lack of contrast agent in that study. Although these studies are comparable to our study in terms of voxel volume, our approach alleviated the need for breath‐holding. Moreover, all these studies report residual B_1_^+^ inhomogeneity as a source of image impairment, which was largely eliminated in our study using phase shimming based on Fourier PE‐DREAM B_1_^+^ maps. As such, these studies could directly benefit from the approach outlined here, and it would be interesting to re‐evaluate the clinical feasibility of these 7 T liver protocols and their performance compared to lower field strengths.

Interestingly, many of the aforementioned papers report the presence of a hyperintense vasculature signal in T_1_‐weighted GRE acquisitions at 7 T. The same effect was also observed in our study. The potential benefit of this effect was shown for intracranial [76, 77, 78, 79] and renal [80, 81] acquisitions, and could likewise be of diagnostic value for non‐enhanced MR angiography in the liver. The possibility of a decreased dosage or complete omission of contrast media at UHF is particularly relevant considering the potential side effects associated with the administration of gadolinium‐based contrast agents [82].

There has been an ongoing debate about the exact origin of this hyperintensity, ranging between the use of low flip angles, differences in T_1_ and T_2_* relaxation times, steady‐state and inflow effects, and utilization of local transmit‐receive coils [74, 77, 83, 84]. In our study, slab‐selective pulses were used for 7 T and 3 T, with identical FOV containing the full abdomen. However, whereas a whole‐body transmit coil was used at 3 T, our 7 T setup utilized a local transmit‐receive fractionated dipole array, which has a more limited sensitive area. Additionally, for 7 T the B_1_^+^ field was explicitly optimized in the liver only, allowing a signal drop‐off elsewhere. On top of that, we found that at 3 T, liver vasculature did not appear brighter than liver tissue, irrespective of flip angle (Ernst angle or lower) or TE. Based on these findings, our study reinforces the hypothesis that the hyperintensity is primarily caused by steady‐state and inflow effects, combined with the utilization of local transmit‐receive coils, rather than low flip angle or differences in relaxation times. When using a transmit body coil (as here for the 3 T scans), flowing spins are pre‐saturated by RF pulses when entering the imaging region, whereas this is not the case when using local excitation [74, 83]. Although not a primary cause in itself [83], the higher T_1_ at 7 T could have a secondary effect when combined with this inflow effect, because saturation of background tissue is amplified, increasing blood‐tissue contrast further. Relatedly, the use of a local transmit‐receive coil also led to reduced aliasing: whereas at 3 T, severe fold‐over of the arms was observed for both participants, mild to no fold‐over was observed at 7 T, likely due to the arms being outside the sensitive coil area. This advantageous lack of fold‐over allows a smaller FOV in the RL‐direction, and hence shorter acquisition time.

The comparison between 3 T and 7 T showed that image sharpness and perceived SNR were comparable, irrespective of flip angle (Ernst angle or lower) or TE. It is important to note that this was achieved, despite 3 T having 3.5 times the number of receive coil elements (28 at 3 T vs. 8 at 7 T), which inherently improves CS reconstruction at 3 T. This higher number of receive channels at 3 T likely compensated for the expected SNR loss at lower field strength, suggesting that a fairer comparison would require equalized coil configurations in future studies. The comparison also revealed a “wavy” artifact that was present at both field strengths. Contrary to the aforementioned discussion about the possible impact of increased B_0_‐effects near the liver dome at 7 T, this finding suggests that this “wavy” artifact throughout the liver may be related to field strength‐independent effects, such as undersampling. This would also explain the absence of these artifacts in FB 4.35 mm (Figure 5), in which virtually no undersampling was employed. Future studies could therefore explore whether tailoring the scan durations (i.e., undersampling factors) further mitigates these artifacts for high‐resolution acquisitions.

Recently a promising, 7 T abdominal MRI technique was published by Maatman et al. [84], using a radial stack‐of‐stars sequence with FID navigators for respiratory gating. 3D liver GRE images were acquired at a higher, anisotropic, resolution (0.8 × 0.8 × 2.0 mm^3^) compared to the isotropic resolution (1.35 mm) used here. However, scan time was longer (13:45 vs. 3:28 min), as no significant undersampling was employed yet. Additionally, our use of (pseudo‐)Cartesian sampling, as opposed to radial stack‐of‐stars, reduced computational complexity by eliminating the need for regridding operations, such as the inverse non‐uniform fast Fourier Transform. Interestingly, the use of Time Interleaved Acquisition of MOdes (TIAMO) optimized for the entire abdomen, rendered B_1_ inhomogeneity‐related impairment in the liver “insignificant” as rated by two radiologists. Still, optimizing for the entire abdomen unavoidably slightly compromised liver homogeneity, as visible in the axial reconstructions, compared with the single‐phase‐shim approach focusing on the liver used here. The increased liver homogeneity in our approach could also partly be due to the application of N4BiasFieldCorrection to reduce receive coil (B_1_^−^) inhomogeneity effects. Also, Maatman et al. used binomial water excitation pulses to effectively suppress subcutaneous fat signal, to avoid off‐resonance blurring of lipid signal. However, this led to a restriction in mean flip angle of ≈2.5° due to SAR constraints. Therefore, they propose a Dixon approach as an alternative fat‐water separation method [85], which would allow significantly higher flip angles. Another advantage of the approach used here is the use of absolute, 3D, all‐channel‐on Fourier PE‐DREAM B_1_^+^ mapping under breath‐holds, acquired in the same respiration phase that was used for image reconstruction, ensuring accurate B_1_^+^ shimming in the liver area. Similarly to our study, Maatman et al. also reported residual artifacts related to motion, B_0_‐inhomogeneity and magnetic susceptibility near air‐tissue interfaces, underlining the room for further improvement. Nevertheless, liver vessel depiction was rated moderate (coronal and sagittal) to good (axial), and, as in our work, they showed vasculature enhancement without the use of a contrast agent, demonstrating the feasibility of their approach. Together, both papers show the feasibility of FB abdominal imaging at 7 T.

## 5 Conclusion

7 T MRI with phase shimming achieves high‐resolution liver imaging with sufficient SNR, while FB scans with respiratory binning minimize motion artifacts. This enables a 1.35 mm isotropic resolution, advancing non‐invasive liver examinations.

## Supporting information


**Figure S1.** Video cycling through coronal slices of the 1.35 mm acquisition in Participant 2.

**Figure S2.** Comparison of 3 T and 7 T liver MRI results for Participant 2, showing one axial and two coronal slices at isotropic resolutions of 1.50 and 1.35 mm. The 3 T scans were acquired using a flip angle (FA1) that provided equivalent liver T_1_‐weighting to 7 T. Red arrows highlight fold‐over artifacts for 3 T, which were not observed for 7 T in this participant. Red arrowheads highlight signal dropouts in the subcutaneous fat (B_0_‐related) and to the contralateral side of the liver (B_1_^+^‐related) for 7 T, which are no issue at 3 T. The posterior bumps for 7 T were caused by the rectangular dipoles in the transmit‐receive coil (yellow asterisks). Contrary to Participant 5 (Figure 8), a minimum TE of 1.45 ms was used, leading to increased chemical shift effect of the 2^nd^ kind at water‐fat boundaries for 3 T, compared to 7 T (blue arrowheads). For clarity, annotations were omitted for the 1.35 mm scans.
**Figure S3.** Comparison of 7 T results (using Ernst angle) with the 3 T results acquired using low flip angle (FA1, leading to equal liver T_1_‐weighting to 7 T) and Ernst angle (FA2), for 1.35 mm resolution in Participant 2 (1.5**×** zoomed‐in with respect to Figure S2). White arrowheads highlight vasculature, appearing bright at 7 T, indistinguishable for 3 T FA1 and dark for 3 T FA2. Contrary to Participant 5 (Figure 9), a minimum TE of 1.45 ms was used, leading to increased chemical shift effect of the 2^nd^ kind at water‐fat boundaries for 3 T, compared to 7 T (blue arrowheads).
**Figure S4.** Coronal single‐slice images reconstructed from data from four respiration phases of the 1.35 mm acquisition in Participant 2. The green dotted ellipses highlight areas of B_1_^+^ inhomogeneity, which are located outside of the liver for all respiration phases for this participant. The same information is shown in a video in Figure S5. SG: self‐gating.

**Figure S5.** Video cycling through coronal single‐slice images reconstructed from data from four respiration phases of the 1.35 mm acquisition in Participant 2. The same information is shown as in the static version of Figure S4.

## Data Availability

The data that support the findings of this study are available from the corresponding author upon reasonable request.
