# Supplementary material for: Compressed Sensing‐Accelerated Free‐Breathing Liver MRI at 7 T
Source: NMR Biomed. 2025 Apr 28;38(6):e70047. doi: 10.1002/nbm.70047 (PMC12038085; doi:10.1002/nbm.70047)
Supplement: Supplementary file 2 — Figure S2. Comparison of 3 T and 7 T liver MRI results for Participant 2, showing one axial and two coronal slices at isotropic resolutions of 1.50 and 1.35 mm. The 3 T scans were acquired using a flip angle (FA1) that provided equivalent liver T1‐weighting to 7 T. Red arrows highlight fold‐over artifacts for 3 T, which were not observed for 7 T in this participant. Red arrowheads highlight signal dropouts in the subcutaneous fat (B0‐related) and to the contralateral side of the liver (B1 +‐related) for 7 T, which are no issue at 3 T. The posterior bumps for 7 T were caused by the rectangular dipoles in the transmit‐receive coil (yellow asterisks). Contrary to Participant 5 (Figure 8), a minimum TE of 1.45 ms was used, leading to increased chemical shift effect of the 2nd kind at water‐fat boundaries for 3 T, compared to 7 T (blue arrowheads). For clarity, annotations were omitted for the 1.35 mm scans. Figure S3. Comparison of 7 T results (using Ernst angle) with the 3 T results acquired using low flip angle (FA1, leading to equal liver T1‐weighting to 7 T) and Ernst angle (FA2), for 1.35 mm resolution in Participant 2 (1.5× zoomed‐in with respect to Figure S2). White arrowheads highlight vasculature, appearing bright at 7 T, indistinguishable for 3 T FA1 and dark for 3 T FA2. Contrary to Participant 5 (Figure 9), a minimum TE of 1.45 ms was used, leading to increased chemical shift effect of the 2nd kind at water‐fat boundaries for 3 T, compared to 7 T (blue arrowheads). Figure S4. Coronal single‐slice images reconstructed from data from four respiration phases of the 1.35 mm acquisition in Participant 2. The green dotted ellipses highlight areas of B1 + inhomogeneity, which are located outside of the liver for all respiration phases for this participant. The same information is shown in a video in Figure S5. SG: self‐gating. [file NBM-38-e70047-s002.docx]

**Supplementary material**

**Compressed Sensing‐Accelerated Free‐Breathing Liver MRI at 7 T**

**Mitra Tavakkoli*, Bobby A. Runderkamp*, Matthijs H.S. de Buck, Gustav J. Strijkers, Michael D. Noseworthy, Aart J. Nederveen, Matthan W.A. Caan and Wietske van der Zwaag**

*Author MT and Author BR contributed equally to this work


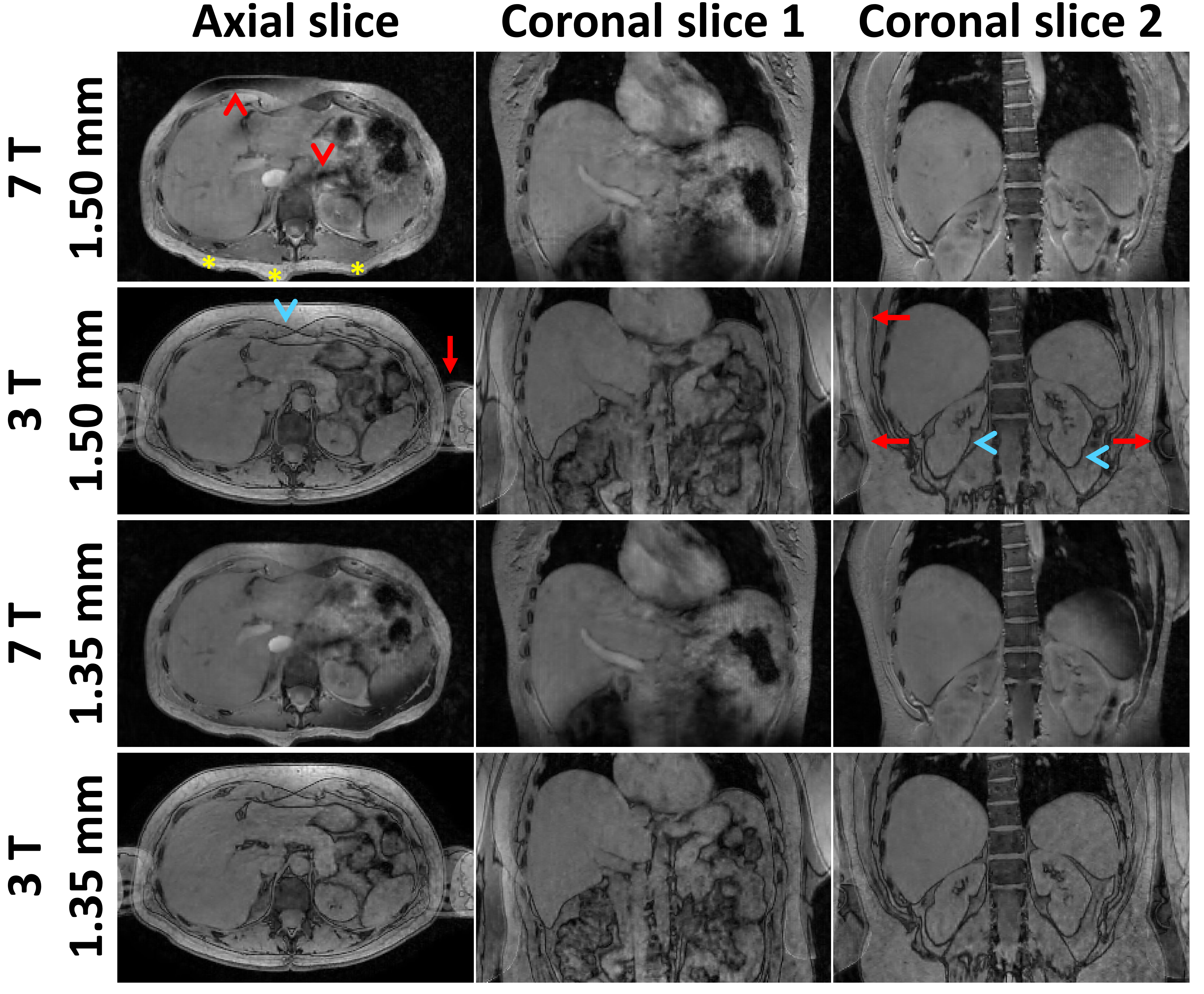


**Figure S2.** Comparison of 3 T and 7 T liver MRI results for Participant 2, showing one axial and two coronal slices at isotropic resolutions of 1.50 and 1.35 mm. The 3 T scans were acquired using a flip angle (FA1) that provided equivalent liver T_1_‐weighting to 7 T. Red arrows highlight fold-over artifacts for 3 T, which were not observed for 7 T in this participant. Red arrowheads highlight signal dropouts in the subcutaneous fat (B_0_‐related) and to the contralateral side of the liver (B_1_^+^‐related) for 7 T, which are no issue at 3 T. The posterior bumps for 7 T were caused by the rectangular dipoles in the transmit‐receive coil (yellow asterisks). Contrary to Participant 5 (Figure 8), a minimum TE of 1.45 ms was used, leading to increased chemical shift effect of the 2^nd^ kind at water‐fat boundaries for 3 T, compared to 7 T (blue arrowheads). For clarity, annotations were omitted for the 1.35 mm scans.


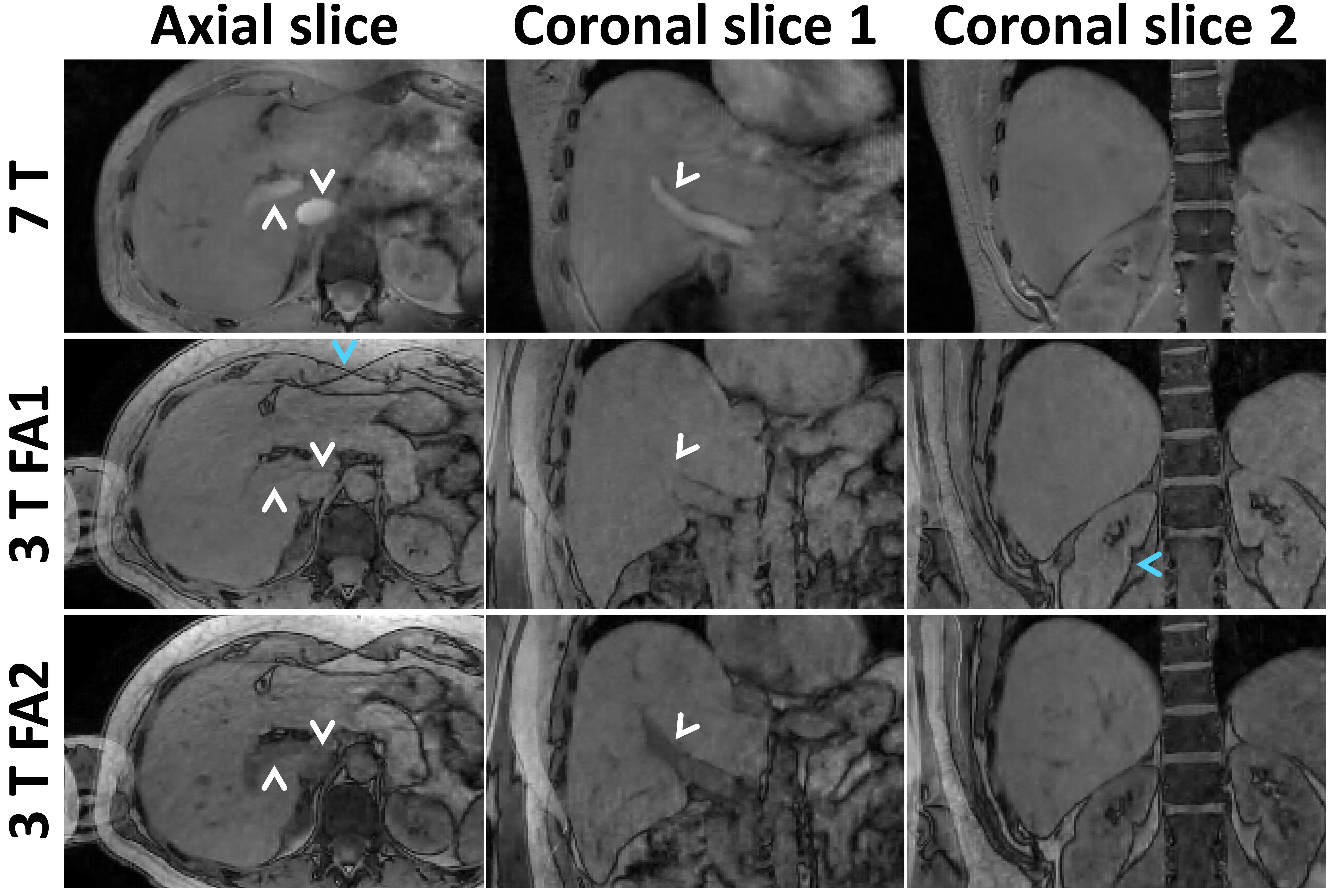


**Figure S3.** Comparison of 7 T results (using Ernst angle) with the 3 T results acquired using low flip angle (FA1, leading to equal liver T_1_‐weighting to 7 T) and Ernst angle (FA2), for 1.35 mm resolution in Participant 2 in (1.5× zoomed‐in with respect to Figure S2). White arrowheads highlight vasculature, appearing bright at 7 T, indistinguishable for 3 T FA1 and dark for 3 T FA2. Contrary to Participant 5 (Figure 9), a minimum TE of 1.45 ms was used, leading to increased chemical shift effect of the 2^nd^ kind at water‐fat boundaries for 3 T, compared to 7 T (blue arrowheads).


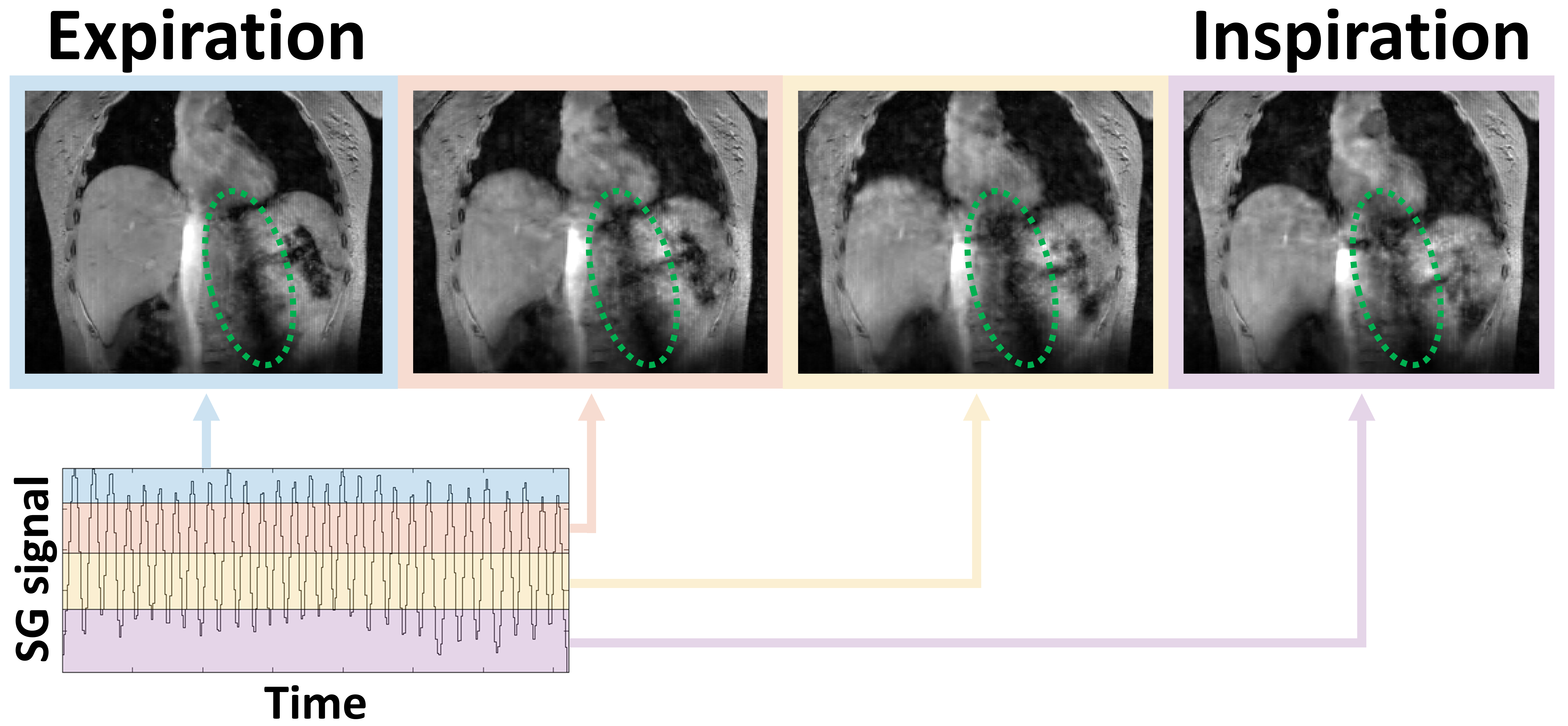


**Figure S4.** Coronal single‐slice images reconstructed from data from four respiration phases of the 1.35 mm acquisition in Participant 2. The green dotted ellipses highlight areas of B_1_^+^ inhomogeneity, which are located outside of the liver for all respiration phases for this participant. The same information is shown in a video in Figure S5. SG: self-gating.
